# Comparison between Two Different Device Models 18 Hz GPS Used for Time–Motion Analyses in Ecological Testing of Football

**DOI:** 10.3390/ijerph17061912

**Published:** 2020-03-15

**Authors:** Jesus Vicente Gimenez, Jorge Garcia-Unanue, Archit Navandar, David Viejo-Romero, Javier Sanchez-Sanchez, Leonor Gallardo, Antonio Hernandez-Martin, Jose Luis Felipe

**Affiliations:** 1 School of Sport Sciences, Universidad Europea de Madrid, 28670 Villaviciosa de Odón (Madrid), Spain; intertato_@hotmail.com (J.V.G.); archit.navandar@universidadeuropea.es (A.N.); david.viejo@universidadeuropea.es (D.V.-R.); Javier.sanchez2@universidadeuropea.es (J.S.-S.); joseluis.felipe@universidadeuropea.es (J.L.F.); 2 IGOID Research Group, Department of Physical Activity and Sport Sciences, University of Castilla-La Mancha, 45071 Toledo, Spain; leonor.gallardo@uclm.es (L.G.); antoniohmds@gmail.com (A.H.-M.)

**Keywords:** football technology, football movement patterns, football external load

## Abstract

Background: The aim of this study was to compare the validity of two different GPS device models used for time–motion analyses in ecological testing of football. Methods: Ten healthy male players from a Spanish university football team participated in this study. The team sport simulation circuit (TSCC) used was based on previous research examining the validity and interunit reliability of different GPS systems. Participants were required to complete eight laps of the TSSC, resulting in a total distance of 1320 m. The GPS units used for the current study were the 18 Hz StatsSport Apex Pro and 18 Hz RealTrack WIMU Pro. Participants were required to wear either of the two GPS units during the test. To establish the construct validity of GPS as a measure of V_max_, timing lights were used as a gold standard. Results: The results clearly suggest that it is not possible to use the same 18 Hz GPS model or interchange it. The measurement can be considered precise when the noise is at least equal to or lower than the smallest worthwhile change. In this case, all standard deviation in measurement error was higher than the smallest worthwhile change. This is due to an inconsistency in the data processing of each trademark. Conclusions: It is important to prevent a club using different GPS trademarks at the same time, since it is not possible to compare in any case any type of result obtained between different trademarks.

## 1. Introduction

The global positioning system (GPS) is a satellite-based navigational technology originally devised for military purposes [1], which was first applied to the field of athlete tracking in 1997 [2] and team sports in 2006 [3]. The recent development of portable GPS units (approximately 8.0 × 4.0 × 1.5 cm and less than 10 g) has permitted a diary application of this technology in sport to provide scientists, coaches and trainers with comprehensive and real-time analysis of on-field player performance during competition or training [1], becoming a standard tool for determining movement patterns during matches and training sessions. Besides this use, GPS technologies have become a standard tool to provide an assessment of training and match load placed on players [4,5,6].

The use of GPS devices has been extended thanks to the fact that they have proved to be valid and reliable devices to describe player movement patterns, speed and distance travelled and number of accelerations and decelerations [7]. Player movement patterns and activity profiles (external loads) can be used in addition to tactical information (spatial x and y coordinates) and physiological responses (internal load) to characterize competitive match play [8]. This information could be a relevant performance indicator because it offers information about a teams’ overall space positioning and distribution principles and how the environmental functional constraints have an effect on the tactical positioning of the team [9]. Therefore, the quantification of different aspects of training and a match’s load is crucial to understand the training process in football [10]. However, the use of GPS during matches is related to establishing performance profiles and provide a framework to design training procedures; its use in training sessions permits a periodization on a daily basis [11].

The intensive daily use of GPS systems has caused multiple companies to develop this type of instrument [12]. On a scientific level, two generations of GPS have been developed. The first one, 1 Hz to 5 Hz systems, has had its reliability and accuracy analysed in previous studies [13,14]. A second generation, with frequencies above 10 Hz, was developed, and validated in previous research, which demonstrated better values of accuracy [15,16,17].

The accuracy of these systems is crucial in team sports such as football because the movement patterns of the players are intermittent in nature [18], with a huge high-intensity load that involves accelerations and decelerations [19], high-intensity runs (14–21 km·h^−1^), very high-intensity runs (21.1–24 km·h^−1^), and sprints (over 24 km·h^−1^) [20]. As a result, research in performance analysis of football suggests that the reaching of peak velocities and the distance covered at very high intensities are critical points [21].

Therefore, the validity and interunit reliability of 1 Hz and 5 Hz GPS units as measures of athlete movement demands have been established previously [22,23]. These studies have shown that the level of GPS interunit reliability decreased when relatively small distances were used for assessment, when sharp changes in direction occurred and when the speed of movement increased. In the same way, the validity and interunit reliability of the research 10 Hz and 15 Hz GPS units have been reported [24,25]. These studies have revealed more valid and reliable values, especially in high-intensity movement patterns, due to the increase in the sampling rate of these devices compared to their predecessors of 1 to 5 Hz.

Due to the high demand for this technology, different brands and suppliers offer GPS systems for team sports. In all cases, they have very similar functionalities, but the treatment of the information may be different. Currently, 10 Hz devices are frequently used in team sport practice and applied studies [26]. However, there are very few studies that analyze the new 18 Hz devices [6,16]. Previous studies have been developed, but there is insufficient evidence of the accuracy of the new 18 Hz devices recently introduced to the market [17,27]. Thus, the aim of this study was to compare the agreement between two different 18 Hz GPS devices used for time−motion analyses in ecological testing of football.

## 2. Materials and Methods

### 2.1. Participants

The study comprised a convenience sample of 10 healthy male players from a Spanish university football team, all of whom volunteered to take part in this study. Players trained as a team 3 days per week for approximately 2 h per training session and competed in one weekly match in the Spanish University League, according to previous studies [6,16,17]. The power of the statistical results ranges from 0.84 to 0.99 for the selected sample. Players were recruited via the football coach, who explained the study to the whole team and that participation was voluntary. The mean (±SD) age, height, body mass and competitive playing age were 21.4 ± 1.1 years; 174.6 ± 3.8 cm; 74.5 ± 6.2 kg; and 10.4 ± 2.9 years, respectively. Each participant provided written informed consent before any testing began, based on the last version of the Helsinki Declaration. This study was approved by the Ethical Committee of the European University of Madrid (CIPI04/2019). To ensure the confidentiality of the players, all performance data were anonymized before analysis.

### 2.2. Procedures

**Figure 1:**
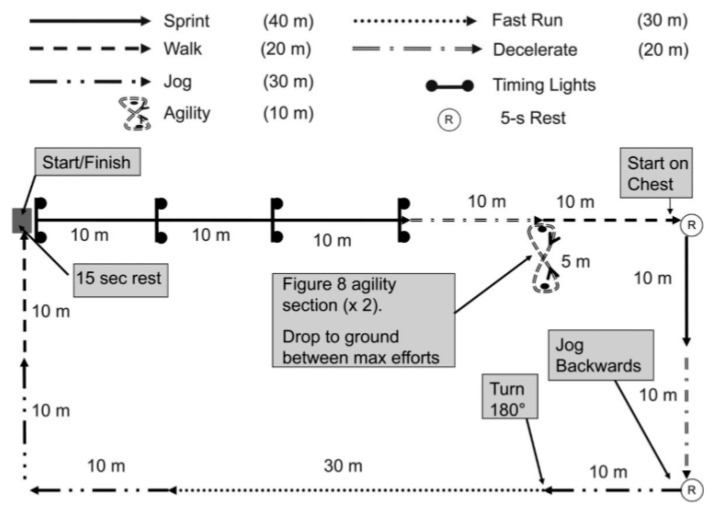
Team sport simulation circuit (TSSC) [25].

The team sport simulation circuit (TSCC) used was based on previous research examining the validity and interunit reliability of different GPS systems in football [22,23,25]. The TSSC was designed to replicate the real movements and physiological requirements of football team patterns, and contained components of standing, walking, jogging, running and sprinting along with accelerations and decelerations. In addition, this protocol has been previously used in other sports such as field hockey [28] or Australian football [29]. Additionally, the TSSC was designed to assess the capabilities of GPS when measuring movement demands at speeds in excess of 20 km·h^−1^. The procedure of this test was described previously by Johnston et al. [25] (Figure 1). Participants were required to complete eight laps of the TSSC, resulting in a total distance of 1320 m, which is comparable with previous examinations into GPS and movement demands [5,30]. It was found that each lap was finished within approximately 1 min, with participants provided with a 15-s rest at the start of each subsequent lap. This meant that testing maintained a similar intensity to team sport matches [31].

The test was carried out on an artificial turf football field in the centre of Spain, on a sunny day, with a temperature of 22–24.5 °C and 12%–25% humidity, during April 2019.

The GPS units used for the current study were 18 Hz StatsSport Apex Pro (firmware: Apex Pro Series software v. 2.0.2.4, STATSports, Newry, N. Ireland) and 18 Hz RealTrack WIMU Pro (firmware: SPRO Software v. 948, Realtrack Systems SL, Almería, Spain). Participants were required to wear one of the GPS units during the test. These units were placed inside separate specially designed garments and positioned so that they sat between the participants’ scapulae, as indicated by Johston et al. [25].

The same variables, according to previous studies [20], were obtained from both systems: total distance (TD, distance covered by the player throughout the test independent from the speed at which players were moving); distance in zone 1 (D_Z1_, distance in metres covered by the player at a speed lower than 4.00 km·h^−1^); distance in zone 2 (D_Z2_, distance in metres covered by the player at a speed of between 4.00 and 7.99 km·h^−1^), distance in zone 3 (D_Z3_, distance in metres covered by the player at a speed of between 8.00 and 13.99 km·h^−1^) distance in zone 4 (D_Z4_, distance covered in metres by the player at a speed of between 14.00 and 20.99 km·h^−1^); distance in zone 5 (D_Z5_, distance in metres covered by the player at a speed of between 21.00 and 23.99 km·h^−1^); distance in zone 6 (D_Z6_, distance in metres covered by the player at a speed equal to or greater than 24.00 km·h^−1^), maximum speed (V_max_, peak height velocity achieved by the player, recorded in km·h^−1^), and average velocity (V_mean_, average velocity by the player throughout the test, recorded in km·h^−1^).

To establish the construct validity of GPS as a measure of V_max_, timing lights (Microgate, Bolzano, Italy) were used as gold standard [32]. Timing lights were placed at the start and at 0, 10, 20, and 30 m marks along the 30 m sprint at the commencement of the TSSC. Average velocity from the 20–30 m split was used to calculate peak speed according to Johnston et al. [25].

Participants were required to complete a familiarity test. They were walked with the researcher through the circuit before testing. Cones and instructional markers were used throughout the TSSC to assist participants in understanding what was required of them and to ensure the circuit was correctly followed. The familiarity session was done on the same football pitch where one of the trial runs was done.

### 2.3. Statistical Analysis

Mean error (Diff) was calculated by deducting Wimu values from the Apex values. To establish the level of agreement between the two devices, the following tests were performed: mean absolute error (MAE), root mean square error (RMSE), product moment correlation (Corr), intraclass correlation coefficient (ICC) of the total agreement, and standard error of the estimate in standardized terms (SEE). Bland–Altman plots with mean error and 95% limits of agreement were also included. Vmax values estimated by Wimu and Apex devices were compared with those obtained by timing gates using Corr, ICC and SEE tests. Furthermore, standard deviation (SD) and the smallest worthwhile change (SWC) were included. The measurement can be considered as useful for precise measurements when the SD is at least equal to or lower than the SWC [33]. The SWC could be defined as 0.2 multiplied by the between-subject SD [34].

## 3. Results

**Figure 2:**
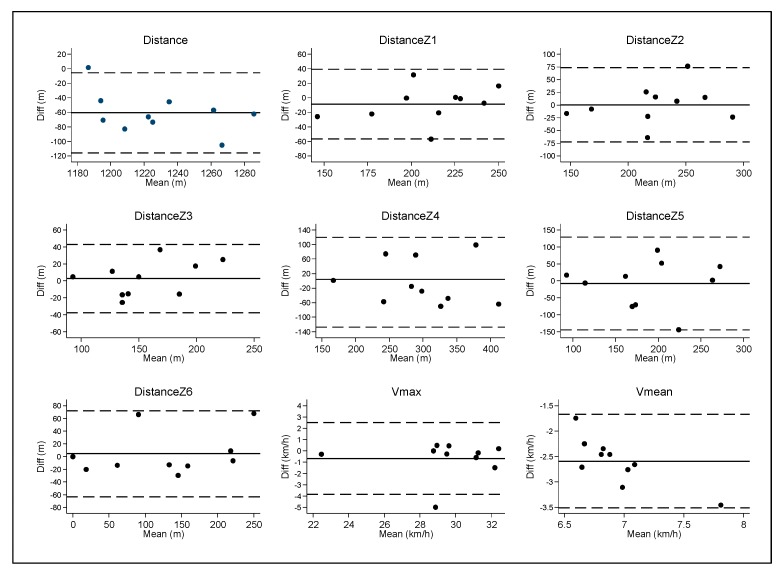
Bland−Altman analysis between Apex and Wimu system in every variable.

Table 1 show the errors measured between Apex vs Wimu systems for the distance and velocity variables in semi-professional football players. The mean error (Diff), and the MAE show irregular values for the distance variables at all speeds, which is above 5% of the actual measured values. SWC is higher than SD of the measurement error (Figure 2). However, ICC values were from moderate and high in distance variables and very high in the high-intensity variable (D_Z6_ and V_max_).

**Figure 3:**
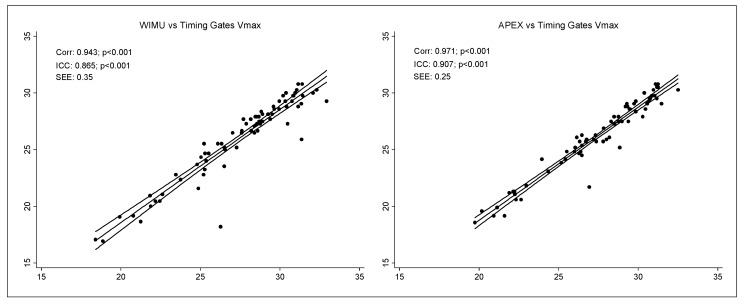
Product–moment Correlation for V_max_ for both systems and the gold standard timing gates.

Figure 3 shows the product−moment correlation between both systems and the gold standard measurement: timing gates in V_max_ values. The Wimu (0.943) and Apex (0.971) systems present nearly perfect values, with high (Wimu = 0.865) and very high (Apex = 0.907) ICC values for V_max_.

## 4. Discussion

The current study aimed to assess the intra-participant reliability of two different GPS device models (Apex and Wimu), both operating at a sampling rate of 18 Hz, respectively, in testing protocol TSSC, used in previous investigations [22,23,25], and the gold standard measurement photocells in a university football team. Both systems have been used and researched in elite football on a regular basis (e.g., Premier League, Serie A or La Liga) [17,35]. Our findings demonstrate that the GPS systems analysed—even in maximal intensity football-specific exercises—accurate position, average speed and peak speed measurements; whether the error margins are acceptable, showing consistent results, depends on the purpose of the analysis. However, neither of the GPS devices could be used within the same football game scenarios or training sessions interchangeably.

The main findings showed that both GPS systems used for the record of zone velocity and maximal running speed have high reliability values and did not present substantial variations in movement patterns. In fact, the validity and agreement showed moderate and very high ICCs for D_Z6_ and V_max_, respectively. This is an important finding, since it contrasts with previous studies [11,36,37] that have detected a low accuracy during high-intensity activities. This may be because these studies have used devices with a low sampling rate (5 Hz). Previous studies [5,6] have highlighted that the sampling rate is a crucial factor associated with the validity and reliability of this technology. Higher sampling frequency devices, such as those used in this study (18 Hz), are more accurate and reliable than previous devices (1–5 Hz and 10–15 Hz) [38]. Based on such evidence, it seems logical to assume that a large sampling rate, such as the one used in this study (18 Hz), resulted in an improvement in the validity of results, especially during the high-intensity values (e.g., V_max_ or D_Z6_) as indicated in previous studies [6,39,40].

Both systems, but specially Apex Pro 18 Hz, demonstrated improved reliability vs. gold standard measurements in the ecological-specific football test. The data obtained confirm that, as the rules of the game are updated, the physical values detected in football players constantly increase [31]. Accordingly, when the V_max_ is reached (>29 km·h^−1^) it is essential to have a technology accurate enough to measure high-intensity actions in both real games and training situations. It may be noted that high-intensity actions are clearly affected by the type or version of data analysis software of each device, and that each trademark has specific analysis algorithms. Buchheit et al. [36] highlighted that the measurement of accelerations and decelerations were large, with a variation of up to 50% by the type or update of the software, while V_max_ actions were affected by 1% and actions >21 km·h^−1^ in a 6% maximum. Given that, our results did not substantially affect the distance covered at high-intensity speed or V_max_, which were designated as the key actions in the quantification of the external workload in elite football [5,41,42].

Thereby, this study demonstrates a high consistency of GPS devices in reliability football situations, especially between high-intensity actions. This demonstrated that both devices may be used by football clubs in routine training sessions and game settings effectively. This situation has been previously demonstrated [43]. However, previous research [44] has shown that the dispersion measures of these devices rise with increasing speed, reaching a 69% coefficient of variation in the high-intensity distance category (>21 km·h^−1^). Thus, one of the most relevant aspects of this research is that these types of devices, with a sampling frequency of 18 Hz, remain reliable even in these types of performance-determining situations in elite football. However, Randers et al. [44] revealed rather large between-system differences during an elite match situation in the determination of the absolute distances covered, implying that any comparison of results using different match analysis systems should be done with caution. Nevertheless, they confirm that different GPS systems were able to detect performance decrements during a football game and can be applied to study development and movement patterns in elite football.

Finally, as a proposal for a future research line, it would be interesting to monitor a professional football team with two devices of different trademarks at the same time in each player. This would provide clear evidence of the variations in results in real-game situations.

## 5. Conclusions

Football coaches and physical trainers should be aware that both 18 Hz GPS systems could be used in real training and competition situations with a high reliability for high-intensity football variables. However, the results clearly suggest that it is not possible to interchange different GPS models. The measurement can be considered precise when the noise is at least equal to or lower than the SWC [33]. In this case, all SDs of measurement errors were higher than the SWC. This is due to an inconsistency in the data processing by each trademark. Thereby, to eliminate inter-unit variation, GPS devices should not be used interchangeably.

## Figures and Tables

**Table 1:** Validity and agreement of the distance and velocity variable.

Variables	Apex		Wimu									
	M	SD	M	SD	Diff	SD	MAE	RMSE	SWC	Corr	ICC	SEE
Total Distance (m)	1197.85	30.23	1258.51	42.12	−60.66	28.17	60.94	66.28	9.47	0.74 *	0.30 *	0.90
Distance_Z1_ (m)	205.16	36.86	213.92	29.19	−8.76	24.45	18.29	24.79	6.53	0.75 *	0.72 *	0.88
Distance_Z2_ (m)	224.24	50.74	223.70	42.52	0.54	37.30	27.52	35.39	9.11	0.69 *	0.71 *	0.69
Distance_Z3_ (m)	157.16	43.59	154.49	35.56	2.67	20.49	17.29	19.62	7.75	0.89 *	0.88 *	0.53
Distance_Z4_ (m)	295.76	74.30	299.71	80.85	−3.95	63.11	52.92	60.01	15.12	0.67 *	0.69 *	1.10
Distance_Z5_ (m)	183.46	68.74	191.66	66.74	−8.21	69.88	51.21	66.8	13.22	0.47	0.49	1.89
Distance_Z6_ (m)	132.08	92.98	127.58	82.85	4.50	34.53	24.08	33.06	17.15	0.93 *	0.93 *	0.40
V_max_ (km·h^−1^)	29.19	2.95	29.85	2.94	−0.66	1.62	0.89	1.67	0.58	0.85 *	0.84 *	0.62
V_mean_ (km·h^−1^)	5.64	0.21	8.23	0.56	−2.59	0.47	2.59	2.63	0.28	0.58	0.02	1.41

* *p* < 0.05. M = Mean; SD = standard deviation; Diff = difference between means; MAE = mean absolute error; RMSE = root–mean–square deviation; SWC = smallest worthwhile change; Corr = product−moment correlation; ICC = intraclass correlation coefficient; EES = standard error of the estimate.
